# Dexamethasone Impairs ATP Production and Mitochondrial Performance in Human Trabecular Meshwork Cells

**DOI:** 10.3390/cimb46090587

**Published:** 2024-09-05

**Authors:** Shane Kennedy, Clayton Williams, Emily Tsaturian, Joshua T. Morgan

**Affiliations:** 1 Department of Molecular, Cell and Systems Biology, University of California-Riverside, Riverside, CA 92521, USA; 2 Department of Bioengineering, University of California-Riverside, Riverside, CA 92521, USA

**Keywords:** trabecular meshwork, glaucoma, dexamethasone, mitochondria

## Abstract

Mitochondrial damage occurs in human trabecular meshwork (HTM) cells as a result of normal aging and in open angle glaucoma. Using an HTM cell model, we quantified mitochondrial function and ATP generation rates after dexamethasone (Dex) and TGF-β2 treatments, frequently used as in vitro models of glaucoma. Primary HTM cells were assayed for metabolic function using a Seahorse XFp Analyzer. We additionally assessed the mitochondrial copy number and the expression of transcripts associated with mitochondrial biogenesis and oxidative stress regulation. Cells treated with Dex, but not TGF-β2, exhibited a significant decrease in total ATP production and ATP from oxidative phosphorylation relative to that of the control. Dex treatment also resulted in significant decreases in maximal respiration, ATP-linked O_2_ consumption, and non-mitochondrial O_2_ consumption. We did not observe significant changes in the level of mitochondrial genomes or mRNA transcripts of genes involved in mitochondrial biogenesis and oxidative stress regulation. Decreased mitochondrial performance and ATP production are consistent with the results of prior studies identifying the effects of Dex on multiple cell types, including HTM cells. Our results are also consistent with in vivo evidence of mitochondrial damage in open-angle glaucoma. Overall, these results demonstrate a decrease in mitochondrial performance in Dex-induced glaucomatous models in vitro, meriting further investigation.

## 1. Introduction

Open-angle glaucoma (OAG) is the second leading cause of irreversible blindness worldwide [1]; intraocular pressure (IOP) is the only known modifiable risk factor for the major OAG subtypes, including pseudoexfoliative glaucoma and primary OAG (POAG) [2,3,4]. The major site of IOP regulation is the trabecular meshwork (TM). As humans age, the trabecular meshwork (TM) and its cells become more fibrotic [3,5], senescent [6], and apoptotic [7,8]; additionally, there is increased oxidative stress [9]. These changes are exacerbated in OAG. However, current frontline therapeutics such as prostaglandin analogues do not target the TM [10]. While one newer therapeutic, netarsudil, does target the TM, there are disadvantages regarding its efficacy compared to that of prostaglandin analogues [11]. Therefore, there is a continuing need to identify novel therapeutic targets in the TM.

In both POAG and normal aging, TM cells suffer from several forms of mitochondrial damage, including damage to mitochondrial proteins [12], in particular complex I [13]. Mitochondrial DNA is known to become damaged as well, manifesting as oxidized bases (e.g., 8-Oxo-2′-deoxyguanosine [14]), the mitochondrial common deletion [14], and a decrease in mitochondrial DNA per cell [14,15]. Mitochondria are a major source of cellular adenosine triphosphate (ATP) [16], mediating other processes such as apoptosis, protein synthesis, and the management of reactive oxygen species (ROS). Mitochondria are also involved in the translation of specific proteins, most notably those which make up parts of the electron transport chain complexes I, III, IV, and V [16]. They are also one of the largest sources of ROS in the cell [17]; this is particularly relevant for the TM, as ROS are a major source of damage in aged or glaucomatous TM [7,13]. Unfortunately, quantitative studies on mitochondrial function and dysfunction in TM are somewhat limited.

One early study conducted on calf TM indicated that approximately 1/4 of the ATP used in these cells comes from oxidative phosphorylation (OxPhos) [18,19]. This study used oxygen consumption, along with biochemical assays, to infer how much ATP was derived from glycolysis versus OxPhos. A more recent study on the role of mitochondrial function in cultured HTM cells found that approximately 1/3 of the ATP in cultured human HTM cells comes from OxPhos [20], broadly consistent with the results of the calf TM study. This study also looked at the impact of dexamethasone (Dex) treatment, which is commonly used to induce glaucomatous phenotypes in HTM cells. Specifically, this study looked at proton leak, spare respiratory capacity, maximal respiration, and non-mitochondrial oxygen consumption and found these measures increased after Dex treatment. Overall, these data support the idea that OxPhos supplies less than half the ATP used by HTM cells. This is consistent with the behavior of several other cell types, including astrocytes [21], mesenchymal stem cells [22], and most cancer cell lines [23]. However, this is not universal, as other cell types can predominantly be powered by OxPhos, including dermal fibroblasts [24] and retinal ganglion cells [25].

In addition to the role of mitochondria in producing ATP, they are also a source of cellular ROS. ROS can be removed enzymatically, for example, through superoxide dismutase 2 (SOD2) and catalase (CATA). SOD2 converts superoxide (O_2_^•−^) into hydrogen peroxide (H_2_O_2_), which is further converted into water (H_2_O) and molecular oxygen O_2_ by CATA [26,27]. Notably, superoxide is known to be produced through both physiological and pathological mitochondrial function [28]. The production of ROS by the mitochondria is estimated to consume several percent of the mitochondrial oxygen in baseline conditions [29], and this is important for healthy cell signaling and regulation [28]. Classically, complex I and III of the electron transport chain are considered primary sources of ROS generation in the mitochondria, although other contributors exist [30,31]. Importantly, disease and cellular stress can increase mitochondrial ROS production [28,31]; while not rigorously studied, increases in mitochondrial ROS are implicated in glaucoma [32]. Beyond antioxidant enzymes, mitochondrial biogenesis itself can be a mitigating response to ROS and mitochondrial dysfunction. The “master regulator” of mitochondrial biogenesis is widely regarded to be the transcription factor peroxisome proliferator-activated receptor-gamma coactivator 1-alpha (PGC-1α) [33,34,35]. This transcription factor is known to be upregulated in response to ROS [36,37]; it is also established that downstream effectors of PGC-1α can mitigate ROS [38]. Other downstream effectors, such as mitochondrial transcription factor A (TFAM), directly activate transcription of the mitochondrial genome [39,40] and further mediate the degradation of damaged mitochondrial DNA (mtDNA) [41].

In the current work, we sought to quantify mitochondrial function in primary HTM cells, including the rate of ATP production and the proportion of ATP from OxPhos versus glycolysis. Specifically, we assessed this after treatment with dexamethasone (Dex) and transforming growth factor β2 (TGF-β2), which are frequently used to induce glaucomatous phenotypes in HTM cells in vitro [20,42,43,44]. We additionally assessed basal respiration, ATP-linked respiration, mitochondrial spare capacity, proton leak, and non-mitochondrial oxygen consumption. Finally, we evaluated the extent to which mtDNA per cell was altered as a result of Dex treatment and assessed the degree to which transcript expression associated with mitochondrial biogenesis and the response to ROS were altered by Dex treatment.

## 2. Materials and Methods

### 2.1. Cell Isolation and Culture

All work involving human tissue was performed in a manner consistent with the Declaration of Helsinki. Primary cultures of HTM cells were isolated from donor human corneoscleral rims deemed unsuitable for transplant (Saving Sight, St. Louis, MO, USA); the researchers had no role in tissue recovery or donor identification. The isolation procedure was performed as previously described [45]. Briefly, the iris and ciliary body were removed from the corneoscleral rim, allowing for dissection of the meshwork. The meshwork was then carefully dissected out of the tissue in 10- to 20-mm segments and placed, with 0.2% Cytodex beads (Sigma-Aldrich, St. Louis, MO, USA), in Dulbecco’s modified Eagle medium/Nutrient Mixture F-12 (50:50; DMEM/F12 with L-glutamine and 15 mM HEPES; Corning, Manassas, VA, USA), supplemented with 10% fetal bovine serum and 1% penicillin/streptomycin (P/S). Cells that migrated out of the tissue were maintained in the aforementioned media and cultured normally. All cells were tested as HTM cells via examining myocilin (*MYOC*) mRNA upregulation after a 3-day 100 nM Dex challenge, as previously described [46]. Equal volume treatments of ethanol were used as vehicle controls, and gene expression was measured as described below. All cell lines used exhibited a four-fold or greater increase in *MYOC* mRNA when treated with Dex.

### 2.2. Quantitative Polymerase Chain Reaction (qPCR)

Briefly, RNA was isolated using the ISOLATE II RNA Mini Kit (Meridian Biosciences, Memphis, TN, USA). DNA was isolated via the Monarch^®^ Genomic DNA Purification Kit (New England Bio Labs, Ipswich, MA, USA). RNA and DNA concentrations were determined via absorbance using the Spectramax M2 Microplate Reader (Biomolecular Systems, San Jose, CA, USA). Reactions for qPCR were performed using the SensiFAST™ SYBR^®^ No-ROX Kit (Meridian Bioscience, Memphis, TN, USA) and the Magnetic Induction Cycler (Biomolecular Systems, Upper Coomera, Queensland, Australia); Glyceraldehyde 3-phosphate dehydrogenase (*GAPDH*) mRNA was used as an endogenous control for *MYOC*, *PGC-1α*, *TFAM*, *SOD2*, and *CATA* mRNA expression. Microglobin DNA was used as a nuclear reference gene for total mtDNA (TMDQ). Primers for *GAPDH*, *MYOC*, *PGC-1α*, *TFAM*, *SOD2*, and *CATA* are shown in Table 1.

### 2.3. Metabolic Assays

Cells were seeded in six wells of a Seahorse XFp miniplate (Agilent Technologies, Santa Clara, CA, USA) at 30,000 to 50,000 cells per well and allowed to attach for 24 h. Three wells were treated with 100 nM of Dex for 3 days, and three wells were treated with an equal volume of ethanol as the vehicle control. Similarly, in plates treated with TGF-β2 (Peprotech, Cranbury, NJ, USA), three wells treated with 1 ng/mL for 3 days were used, along with an equal volume of water as the vehicle control. Tissues from five donors were used, with ages/sexes of 71 M, 71 F, 73 F, 52 M, 63 F, and 56 M. Cells were used up to passage 6 in all cases. All data presented are biological replicates consisting of multiple donors. Due to the limitations of primary cells, not all donor strains could be used for the experiments.

For all Seahorse assays, a biological replicate consisted of cells from a single donor plated in triplicate for experimental and vehicle control treatments; additional wells on the Seahorse miniplates were left empty to be used as negative controls for analysis. Each assay was repeated in separate runs, with different donor cells as biological replicates. After 3 days, the cell metabolic activity was assayed via a Seahorse XFp (Agilent Technologies, Santa Clara, CA, USA) using either the Mito Stress Test Kit or the Real-Time ATP Rate Assay, according to manufacturer instructions. One hour prior to the assays, the cell growth media was removed, replaced with pre-warmed Seahorse assay media (Agilent Technologies), and the media was then incubated for ~45 min at 37 °C without CO_2_ to allow the assay media to reach the proper pH.

**Figure 1:**
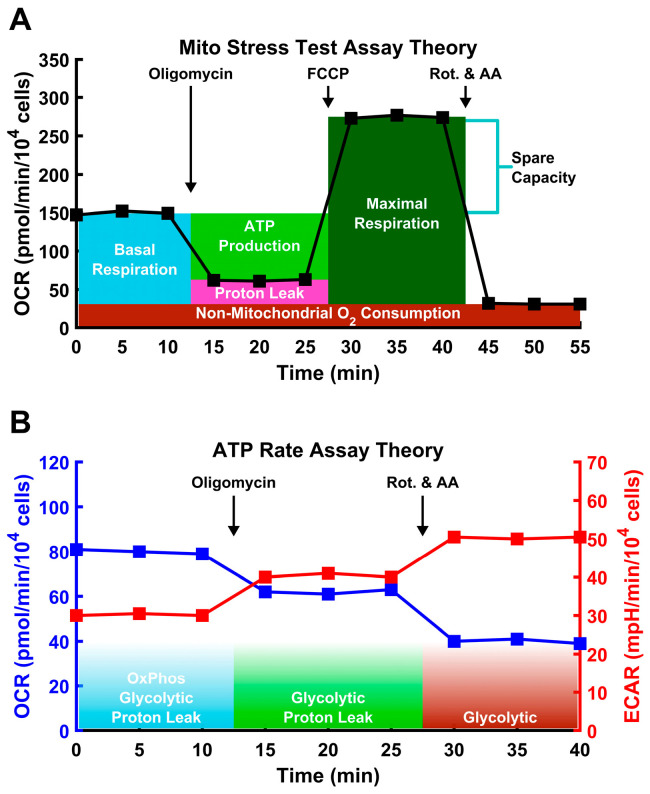
Schematic of Seahorse assays used in this study. In both plots, each square marks a measurement spaced by 5 min. The numbers are representative, not experimental. (**A**) In the Mito Stress Test, the O_2_ consumption rate (OCR) is measured via the pharmacological disruption of the mitochondria to measure specific aspects of mitochondria performance, including maximal respiration induced by the protonophore FCCP. (**B**) In the ATP Rate Assay, OCR and extracellular acidification rate (ECAR) are both measured, allowing for accurate assessment of both oxidative and glycolytic ATP generation. Additionally, FCCP is not used, as this can disrupt the calculation of the glycolysis, as described in the text.

Briefly, the Mito Stress Test works via a sequential injection of 1.5 μM Oligomycin, 2 μM FCCP, and 0.5 μM Rotenone/Antimycin A into the culture wells (Figure 1A). During each 15 min step, the oxygen consumption rate (OCR) and the extracellular acidification rate (ECAR) are measured three times. After an equilibration period of 20 min, basal OCR and ECAR readings are obtained to establish the basal respiration rate. Following the addition of oligomycin, an ATP synthase inhibitor, all ATP synthesis-linked respiration is halted, and the remaining OCR is due to proton leak and non-mitochondrial oxygen consumption. Following the addition of FCCP, a protonophore mitochondrial respiration is uncoupled from ATP synthase, forcing mitochondrial respiration to its maximum capacity. Following addition of Rotenone and Antimycin A (respiratory complex I and III inhibitors, respectively), the electron transport chain is completely disabled, and only non-mitochondrial sources of oxygen consumption remain. The OCR of different sources can then be reconstructed. The OCR devoted to ATP production can be calculated as the difference between the basal OCR and OCR after oligomycin treatment. The final readings provide the non-mitochondrial OCR. Combined with the second set of readings, under oligomycin treatment, OCR associated with proton leak can be determined. The maximum mitochondrial OCR can be determined from the readings after FCCP addition, minus non-mitochondrial OCR. Finally, spare mitochondrial capacity can be determined as the difference between the maximum and basal rates.

The cells were also analyzed via the Agilent ATP Rate Assay. This assay follows a similar treatment pattern to that of the Mito Stress Test, except that it does not include the FCCP treatment step (Figure 1B). In addition to calculating ATP-linked oxygen consumption, this assay also calculates the amount of ATP derived from glycolysis. This cannot be accurately achieved with the Mito Stress Test, as FCCP likely disrupts glycolysis through cytosolic acidification [51,52,53]. Under Rotenone/AA treatment, the electron transport chain is shut down and does not generate CO_2_. Under this condition, the only contributor to ECAR is glycolysis, and it is possible to determine the extent to which glycolysis contributes to ATP production.

Immediately after both assays, the wells were stained with 16 mM of Hoescht 33342 in PBS for 1 h, and each well was completely imaged on a Leica DMI8 microscope (Leica Microsystems, Buffalo Grove, IL, USA). The cell number in each well was quantified via Hoescht labeling using a custom script (MATLAB 2020a; Mathworks, Natick, MA, USA). Briefly, the images from each well were stitched into a single image of the complete well using the phase correlation method [54], and noise was removed through median and top-hat filtering. The images were binarized, and the nuclei were counted. The nuclear count accuracy was checked in the image sub-regions by a masked observer. The OCR and ECAR readings were normalized to the number of cells. ATP production rates were calculated as described [53] using Seahorse Wave Desktop Software™ Version 2.6.3.5.

### 2.4. Statistical Analysis

Values in the text are reported as the mean ± standard error. Paired sample *t*-tests were used to assess the significance of basal respiration, non-mitochondrial oxygen consumption, proton leak, ATP-linked respiration, maximal respiration, and spare capacity, in both absolute and relative terms. The ATP from glycolysis and OxPhos was also assessed via paired-sample *t*-test, both proportionally and in absolute terms as well. A critical value of 0.05 was used in all cases to assess significance, but individual *p*-values are reported throughout. All statistical tests were conducted using RStudio (Version 1.4.1717). Reduced data used to prepare the figures are provided in Supplementary Table S1.

## 3. Results

### 3.1. Dexamethasone Treatment Decreases Relative Mitochondrial ATP Generation

**Figure 2:**
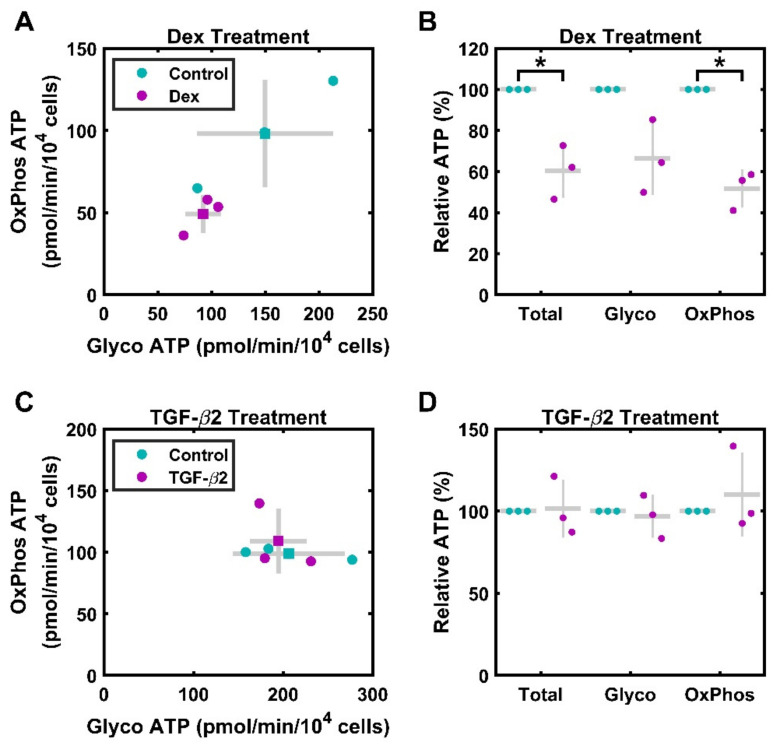
Contributions of oxidative phosphorylation and glycolysis to ATP production. Primary HTM cells were assayed using the Seahorse ATP Rate Assay. (**A**) HTM cells treated with 100 nM Dex for 3 days (magenta) exhibit reduced ATP production compared to that of the control (cyan). Both OxPhos and glycolysis are reduced. ● represent individual experiments; ■ represent means; gray lines represent standard deviation error bars on both axes. (**B**) When quantified relative to control ATP generation, 100 nM Dex treatment for 3 days results in significant decreases in total and OxPhos ATP generation. Control data (normalized to 100%) is included for visual reference. ● represent individual experiments; mean and standard deviation error bars are represented by gray lines. (**C**) HTM cells treated with 1 ng/mL TGF-β2 for 3 days (magenta) exhibit similar ATP production rates compared to those of the control (cyan). ● represent individual experiments; ■ represent means; gray lines represent standard deviation. (**D**) When quantified relative to control ATP generation, 1 ng/mL TGF-β2 treatment for 3 days does not result in significant differences in ATP generation. Control data (normalized to 100%) are included for visual reference. ● represent individual experiments; mean and standard deviation error bars are represented by gray lines. * indicates *p* < 0.05.

HTM cells treated with 100 nM Dex for 3 days were assayed using the ATP Rate Assay, which measures O_2_ consumption and acidification, allowing for the derivation of ATP production rates from oxidative and glycolytic sources. Cells from three separate donors were used, and each data point represents cells of a single donor, measured in technical triplicates (Figure 2A,B). In absolute terms, there was a nonsignificant decrease in ATP production from glycolysis, 92.2 ± 9.5 pmol/min/10^4^ cells, compared to that of the vehicle control, 150.0 ± 36.5 pmol/min/10^4^ cells; (*p* = 0.147). This was also the case for ATP produced by the mitochondria, which under Dex treatment was lower, 49.1 ± 6.6 pmol/min/10^4^ cells, than under vehicle control, 98.0 ± 18.9 pmol/min/10^4^ cells (*p* = 0.077). To mitigate the significant donor-to-donor variability, we also assessed relative production. There was a statistically significant decrease in overall ATP production, with Dex treated cells producing 60% ± 7.6% (*p* = 0.035) as much ATP as that produced in the control. This decrease was driven by declining OxPhos-based ATP production, although glycolysis may also play a role in this process. ATP from OxPhos was significantly reduced to 51% ± 5.4% (*p* = 0.012) that of the control, while ATP from glycolysis was 66.5% ± 10% that of the control, although this was not significant (*p* = 0.083). The ratio of ATP coming from glycolysis versus OxPhos increased nonsignificantly, at 1.50 ± 0.15 to 1.90 ± 0.21 (*p* = 0.133).

### 3.2. TGF-β2 Does Not Significantly Alter ATP Production

In a separate ATP Rate Assay experiment, HTM cells were treated with 1 ng/mL TGF-β2 for 3 days (Figure 2C,D). In contrast to Dex treated cells, cells treated with TGF-β2 did not show significant changes in the measured metabolic parameters. Overall ATP production was 303.9 ± 14.9 pmol/min/10^4^ cells for treated cells compared to 305.3 ± 33.9 pmol/min/10^4^ cells (*p* = 0.967) for untreated cells. ATP generation from glycolysis was 194.7 ± 36.1 pmol/min/10^4^ cells for treated cells; production from control cells was 206.4 ± 18.3 pmol/min/10^4^ cells (*p* = 0.586). Similarly, ATP generation from OxPhos was 109.1 ± 15.3 pmol/min/10^4^ cells for treated cells; production from control cells was 98.9 ± 2.6 pmol/min/10^4^ cells (*p* = 0.561). In relative terms, there was no significant difference in total ATP or in ATP from each source. Total ATP, ATP from glycolysis, and ATP from mitochondria were 101.5% ± 10% (*p* = 0.900), 96% ± 7% (*p* = 0.724), and 110% ± 14% (*p* = 0.561) compared to the rates for the controls. Overall, the ratio of ATP coming from glycolysis versus OxPhos is stable, at 2.1 ± 0.4 in the controls and 1.9 ± 0.4 in the treated cells (*p* = 0.306).

### 3.3. Dexamethasone Impairs Mitochondrial Function

Next, we utilized Mito Stress tests to gain further insight into the impacts of Dex on mitochondrial function. Unlike the ATP Rate Assay, the Mito Stress test uncouples the electron transport chain from ATP production using the protonophore FCCP. This allows for measurement of the maximal respiratory capacity of the mitochondria, but can corrupt measures of glycolysis [51,52,53]. Basal, maximal, ATP-related, and non-mitochondrial O_2_ consumption were measured, along with coupling efficiency and proton leak. In Table 2, the overall results are presented in both absolute terms and as results relative to the control; *p* < 0.05 is denoted with an * in the last column.

### 3.4. Dexamethasone Does Not Result in a Significant Decrease in mtDNA per Cell

**Figure 3:**
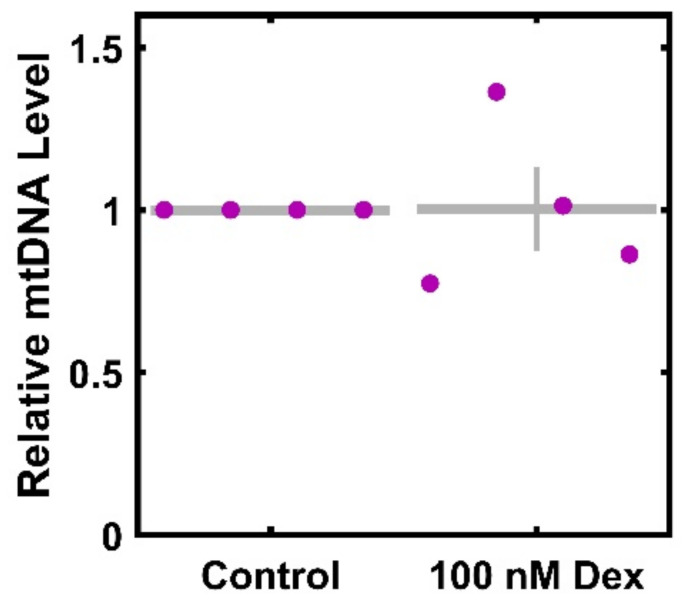
mtDNA levels per cell in response to Dex treatment. HTM cells from four donors were treated with 100 nM Dex for 3 days, and the amount of mtDNA was determined. Control data (normalized to 1) is included for visual reference. ● represent individual experiments; mean and standard deviation error bars are represented by gray lines. There was no significant change in mtDNA levels with Dex treatment.

As reduced ATP generation and maximal consumption could result from a decreased number of mitochondria per cells, we sought to ascertain the extent to which Dex altered mtDNA per cell. There is no significant decrease in mtDNA per cell after Dex treatment (Figure 3), with levels at 100.3% ± 13% of the control (*p* < 0.9807).

### 3.5. Dexamethasone Does Not Result in a Significant Change in ROS-Related mRNA Transcripts

**Figure 4:**
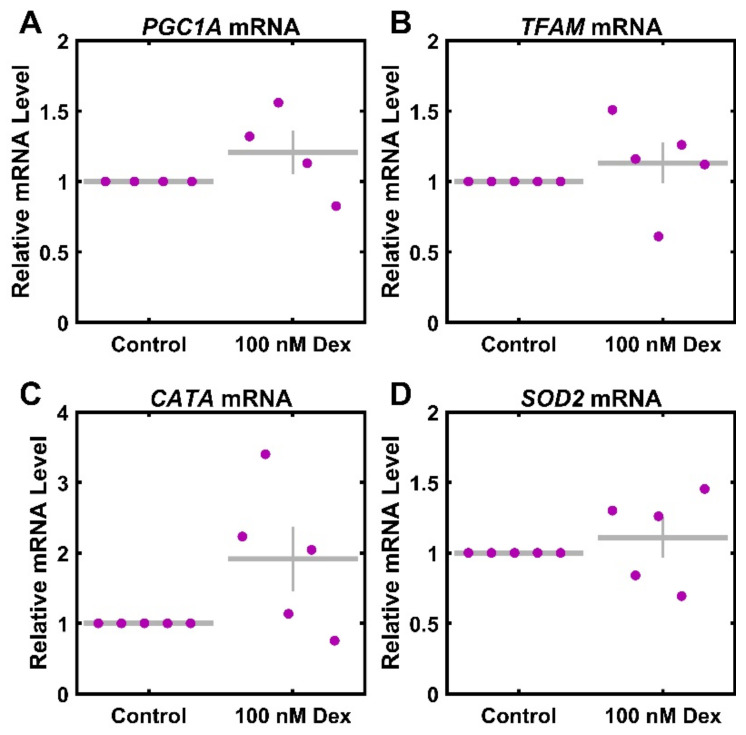
Reactive oxygen species and mitochondrial associated mRNA transcript response to Dexamethasone. Primary HTM cells were treated with 100 nM Dex for 3 days and assayed for (**A**) PGC1A (*n* = 4), (**B**) TFAM (*n* = 5), (**C**) CATA (*n* = 5), and (**D**) SOD2 (*n* = 5) mRNA levels. Control data (normalized to 1) are included for visual reference. ● represent individual experiments; mean and standard deviation error bars are represented by gray lines. There were no significant changes with Dex treatment.

Lastly, as the observed mitochondrial functional changes may impact mitochondrial biogenesis or ROS handling in the cell, we sought to ascertain the extent to which Dex treatment influences the levels of mRNA transcripts as related to these important systems. The relative expression of transcripts for PGC1A and TFAM was used to assess the regulators of mitochondrial biogenesis. The relative expression of transcripts for SOD2 and CATA was used to assess the regulators of ROS. There was no significant change in mRNA levels of PGC1A (Figure 4A) and TFAM (Figure 4B), with levels at 121% ± 16% (*p* = 0.227) and 113% ± 15% (*p* = 0.396) of the control. Further, there was no significant change in either CATA (Figure 4C) or SOD2 (Figure 4D), with levels at 191% ± 46% (*p* = 0.084) and 111% ± 15% (*p* = 0.474) of the control.

## 4. Discussion

Consistent with the results of prior work on calf TM [19] and cultured HTM [20] cells, we showed that cultured HTM cells produce approximately 2/3 of their ATP from glycolysis, with the rest coming from oxidative phosphorylation (Figure 2). The controls for each of the six strains tested exhibit a total ATP production ranging from 151.8–371.0 pmol/min/10^4^ cells. In absolute terms, overall ATP production rates in HTM cells were higher than those of many cell cancer cell lines [55], such as ovarian cancer cell lines OVCAR and NCI/ADR-RES, with total ATP production rates of roughly 40 pmol/min/10^4^ cells and 60 pmol/min/10^4^ cells, respectively [56]. However, the absolute rates are similar to those observed in prior studies of human skin fibroblasts [24] and mouse embryonic fibroblasts [57], which display mean total ATP production rates of approximately 360 pmol/min/10^4^ cells and 250 pmol/min/10^4^ cells, respectively. However, these cell types, unlike HTM cells, obtain a majority of their ATP from mitochondria. In relying on glycolysis, HTM cells are more similar to astrocytes [21], mesenchymal stem cells [22], and many cancer cell lines [23].

Notably, the absolute ATP levels in this study were moderately lower than those reported by Graybeal et al., which showed a total ATP production rate of the control cells between approximately 220 and 950 pmol/min/10^4^ cells [20]. These differences could be attributed to a variety of factors. In this study, there were substantial differences in ATP production among cells of different donors, as shown in the absolute production rates (Figure 2A,C, Table 2). Differences in ATP production between primary cell strains were also reported by Graybeal et al., with results exhibiting approximately order of magnitude differences between the lowest and highest basal rates. While primary cell differences likely account for some of the difference between the studies, differences in seeding density, media, and the experimental timeline could also contribute to these variations.

Overall, we found that Dex-treated cells showed a significant decline in relative ATP production, driven by a significant decrease in OxPhos and a nonsignificant decline in glycolysis. For the three strains tested, Dex treatment resulted in a decrease in both overall ATP production and in OxPhos (Figure 2). By comparison, cells treated with TGF-β2 showed no consistent change in ATP sources, although both Dex and TGF-β2 are associated with induction of a glaucomatous phenotype. This indicates that the mitochondrial effects of Dex, as well as potential implications for glaucoma, are specific to Dex and its related signaling. This may be due to the direct effects on mitochondrial gene regulation, as mitochondrial genes, including those for respiratory complexes, likely exhibit glucocorticoid response elements [58,59].

Further, these findings are consistent with those presenting the effects of Dex in other tissue models, where Dex treatment has been shown to reduce mitochondria and mitochondrial performance [60,61,62]. In particular, one study showed that Dex treatment can lead to a decrease in both respiration and ATP production in mitochondria isolated from rat livers [62]. One report evaluating total ATP levels in C2C12 cells, a myoblast cell line, showed a decrease in total ATP levels after Dex treatments [61]. Another report showed similar results after a 48 h treatment with 1 µM Dex in 3T3-L3 adipocyte cells [60]. Interestingly, this report also showed increased DNA damage in the form of reduced long amplicon qPCR fragments of mitochondrial DNA, an indicator of mitochondrial damage. Reduced mitochondrial ATP production is also consistent with the observed (but nonsignificant) increase in proton leak (Table 2). Proton leak describes the quantity of protons that pass across the inner mitochondrial membrane without going through ATP synthase. This is consistent with the results of prior studies using rat skeletal muscle, where Dex treatment increased proton leak [63]. It is also consistent with a report showing a decrease in the performance of mitochondrial dehydrogenase in HTM cells after DEX treatment [64].

However, these findings are in contrast with those of Graybeal et al., who showed an increase in OxPhos ATP production after Dex treatment of primary HTM cells, as well as an increase in ATP production from glycolysis [20]. There are a number of technical differences between the experiments that may contribute to these differences. Most prominently, Graybeal et al. used a 5-day Dex time course, in comparison to our 3-day treatment; it is known that the effects of glucocorticoids can vary with time in HTM cells [65,66]. This behavior has also been observed in other cell types, e.g., glucocorticoids like Dex can induce mitochondrial biogenesis [67,68,69]; this biogenesis may occur at a timescale longer than 3 days. For example, a mouse model of glucocorticoid-induced glaucoma suggested that a 12-day Dex treatment increased the number of mitochondria in HTM cells [70]. Further, Graybeal et al. used the Mito Stress test, which employs FCCP to uncouple respiration from ATP production. This method can result in changes in the apparent glycolytic ATP production rate; FCCP, a component of the Mito Stress Test, has been shown to decrease glycolysis [51,52,53]. While the exact mechanisms remain unclear, it has been speculated that FCCP leads to a leakage of protons into the cytosol, acidifying it and reducing the function of the pH-sensitive enzymes in glycolysis [52]. Further, another study by Watanabe et al. showed small decreases in basal OCR in response to 250 nM Dex and 5 ng/mL TGF-β2 [71]. While the results presented here generally align with those in prior reports employing many cell types and varying experimental approaches, the variability in the published numbers highlights the importance of experimental context in regards to the effects of Dex.

Our results show a statistically significant decrease in maximal respiration after Dex treatment. This is consistent with a decrease observed in immortalized HTM cells noted by Watanabe et al. [71]. The spare capacity of primary HTM cells, as measured in the current study, ranged from 441.0% to 598.3%, with a mean of 541.4% ± 30.0%. This is consistent with the order of spare respiratory capacity for primary HTM cells reported by Graybeal et al., which displayed values ranging from approximately 160% to 437.5% [20], and higher than the approximately 50% spare respiratory capacity observed in immortalized HTM cells by Watanabe et al. [71]. As a comparison, several studies have reported endothelial cell types with a spare respiratory capacity of approximately 325% [72] or 362% [73]. It is currently unknown whether or not the relatively large spare respiratory capacity of HTM cells exerts a physiological effect; however, the loss of spare respiratory capacity has been linked to aging and disease in other systems [74]. Further studies, especially studies on ex vivo human tissue, are warranted.

As decreases in ATP generation observed with Dex treatment could be related to decreases in mitochondrial number per cell, we used the expression of mtDNA relative to nuclear genomes as a measure for mitochondrial count per cell [15]. We did not observe any decline (Figure 3), suggesting that the functional defects are due to mitochondrial dysfunction and not to gross mitochondrial loss. However, it is important to note that due to the limited number of donors, strong conclusions cannot be drawn, and there is a need for larger studies. Consistent with this negative finding, we also observed no change to *PGC1A* and *TFAM* (Figure 4), two key regulators of mitochondrial biogenesis [33,35,39,40]. As cellular ROS are both regulators of and are regulated by mitochondrial function [75], we also tested the expression of key ROS scavengers *SOD2* and *CATA* [26,30], and found no differences. Again, it is important to note that the strength of these conclusions is limited by the sample size. While more detailed or larger molecular studies are needed, these findings are consistent the observation that the functional effects of Dex treatment are not mediated by transcriptional changes to biogenesis or ROS regulation.

As noted above, the primary limitation of these studies is the limited sample population. With the high donor variability in regards to primary cell culture, small changes in population averages or within subpopulations would not be detected. Additionally, these studies were primarily descriptive, with no molecular mechanism identified.

## 5. Conclusions

In conclusion, we have presented direct measurements of mitochondrial function in primary HTM cells, utilizing both ATP Rate Assays and Mito Stress Tests via a Seahorse XFp Analyzer. In absolute numbers, our findings are broadly consistent with those of prior studies employing similar cell types, generally, and primary HTM, specifically. Additionally, we found that TGF-β2 treatment did not significantly alter mitochondrial function, while Dex treatments reduced ATP generation and impaired mitochondrial function. These in vitro studies add additional quantitative data regarding metabolism in TM biology and reveal Dex-specific impacts on mitochondrial function in HTM cells. When combined with the results from the available literature, these findings motivate further quantitative studies on the regulation of mitochondria in OAG and OAG models and further support mitochondrial function as a potential therapeutic target.

## Figures and Tables

**Table 1:** Primers used for qPCR.

Gene	Forward Sequence	Reverse Sequence	Reference
*GAPDH*	ACAGTCAGCCGCATCTTCTT	GCAGGAGGCGTTGTCATT	[47]
*MYOC*	GGAAGAGAAGAAGCGACT	ATAAACTGGCTGATGAGGTC	[48]
*PGC-1α*	AAACAGCAGCAGAGACAAATGC	TTGGTTTGGCTTGTAAGTGTTGTG	[49]
*TFAM*	TGTTCACAATGGATAGGCAC	TCTGGGTTTTCCAAAGCAAG	[49]
*SOD2*	CTGGACAAACCTCAGCCCT	CTGATTTGGACAAGCAGCAA	[49]
*CATA*	TGGAAAGAAGACTCCCATCG	CCAGAAGTCCCAGACCATGT	[49]
*BGLOB*	GGTGAGTCTATGGGACGCTT	GATCCTGAGACTTCCACACTGA	^a^
TMDQ	CCATCTTTGCAGGCACACTCATC	ATCCACCTCAACTGCCTGCTATG	[14]

^a^ Designed using Primer-BLAST [50].

**Table 2:** Mitochondrial function. Summary of mitochondrial functional parameters after 3-day treatment with 100 nM Dex or vehicle control.

Parameter Name	Control	100 nM Dex	Units	*p*-Value
Mitochondrial O_2_ Basal Consumption	21.9 ± 3.7	16.7 ± 4.1	$\frac{pmol}{min\cdot 10^{4}cells}$	0.0077 *
Relative	100	74.2 ± 10.6	%	0.052
Mitochondrial O_2_ Maximal Consumption	120 ± 26.8	74.9 ± 17.2	$\frac{pmol}{min\cdot 10^{4}cells}$	0.048 *
Relative	100	62.1 ± 5.6	%	0.007 *
O_2_ Consumption for ATP	19.0 ± 3.8	12.8 ± 3.7	$\frac{pmol}{min\cdot 10^{4}cells}$	0.006 *
Relative	100	62 ± 4.8	%	0.032 *
Spare Respiratory Capacity	98.39 ± 23.1	58.3 ± 13.2	$\frac{pmol}{min\cdot 10^{4}cells}$	0.065
Relative (to Basal)	541.4 ± 30.0	459.8 ± 13.8	%	0.130
Relative (to Control)	100	85.4 ± 5	%	0.102
Coupling Efficiency	86.1 ± 3.0	75.7 ± 5.0	%	0.088
Relative	100	87.8 ± 7.1	%	0.097
Proton Leak	2.8 ± 0.2	3.8 ± 0.6	$\frac{pmol}{min\cdot 10^{4}cells}$	0.219
Relative	100	138.0 ± 31	%	0.350
Non-Mitochondrial O_2_ Consumption	22.8 ± 4.0	17.0 ± 3.0	$\frac{pmol}{min\cdot 10^{4}cells}$	0.04 *
Relative	100	74.2 ± 5.0	%	0.012 *

* indicates *p* < 0.05.

## Data Availability

The original contributions presented in the study are included in the article/Supplementary Materials; further inquiries can be directed to the corresponding author.

## Supplementary Materials

The following supporting information can be downloaded at: https://www.mdpi.com/article/10.3390/cimb46090587/s1, Table S1: SI_Data.

## References

[1] Tham Y.-C., Li X., Wong T.Y., Quigley H.A., Aung T., Cheng C.-Y. (2014). Global prevalence of glaucoma and projections of glaucoma burden through 2040: A systematic review and meta-analysis. Ophthalmology.

[2] Boland M.V., Quigley H.A. (2007). Risk factors and open-angle glaucoma: Classification and application. J. Glaucoma.

[3] Quigley H.A. (2011). Glaucoma. Lancet.

[4] Stamer W.D., Acott T.S. (2012). Current understanding of conventional outflow dysfunction in glaucoma. Curr. Opin. Ophthalmol..

[5] Zhavoronkov A., Izumchenko E., Kanherkar R.R., Teka M., Cantor C., Manaye K., Sidransky D., West M.D., Makarev E., Csoka A.B. (2016). Pro-fibrotic pathway activation in trabecular meshwork and lamina cribrosa is the main driving force of glaucoma. Cell Cycle.

[6] Liton P.B., Challa P., Stinnett S., Luna C., Epstein D.L., Gonzalez P. (2005). Cellular senescence in the glaucomatous outflow pathway. Exp. Gerontol..

[7] Saccà S.C., Pulliero A., Izzotti A. (2015). The dysfunction of the trabecular meshwork during glaucoma course. J. Cell. Physiol..

[8] Aktas Z., Karaca E.E., Gonul I.I., Hasanreisoglu M., Onol M. (2013). Apoptosis in the iris and trabecular meshwork of medically treated and untreated primary open angle glaucoma patients. Int. J. Ophthalmol..

[9] Caballero M., Liton P.B., Challa P., Epstein D.L., Gonzalez P. (2004). Effects of donor age on proteasome activity and senescence in trabecular meshwork cells. Biochem. Biophys. Res. Commun..

[10] Schmidl D., Schmetterer L., Garhöfer G., Popa-Cherecheanu A. (2015). Pharmacotherapy of glaucoma. J. Ocul. Pharmacol. Ther. Off. J. Assoc. Ocul. Pharmacol. Ther..

[11] Mehran N.A., Sinha S., Razeghinejad R. (2020). New glaucoma medications: Latanoprostene bunod, netarsudil, and fixed combination netarsudil-latanoprost. Eye.

[12] Zhao J., Wang S., Zhong W., Yang B., Sun L., Zheng Y. (2016). Oxidative stress in the trabecular meshwork (Review). Int. J. Mol. Med..

[13] He Y., Leung K.W., Zhang Y.-H., Duan S., Zhong X.-F., Jiang R.-Z., Peng Z., Tombran-Tink J., Ge J. (2008). Mitochondrial complex I defect induces ROS release and degeneration in trabecular meshwork cells of POAG patients: Protection by antioxidants. Investig. Ophthalmol. Vis. Sci..

[14] Izzotti A., Longobardi M., Cartiglia C., Saccà S.C. (2011). Mitochondrial damage in the trabecular meshwork occurs only in primary open-angle glaucoma and in pseudoexfoliative glaucoma. PLoS ONE.

[15] Izzotti A., Saccà S.C., Longobardi M., Cartiglia C. (2010). Mitochondrial damage in the trabecular meshwork of patients with glaucoma. Arch. Ophthalmol..

[16] Chan D.C. (2020). Mitochondrial Dynamics and Its Involvement in Disease. Annu. Rev. Pathol..

[17] Breitenbach M., Rinnerthaler M., Hartl J., Stincone A., Vowinckel J., Breitenbach-Koller H., Ralser M. (2014). Mitochondria in ageing: There is metabolism beyond the ROS. FEMS Yeast Res..

[18] Epstein D.L., Anderson P.J. (1981). In vitro biochemistry of trabecular meshwork. Vis. Res..

[19] Anderson P.J., Wang J., Epstein D.L. (1980). Metabolism of calf trabecular (reticular) meshwork. Investig. Ophthalmol. Vis. Sci..

[20] Graybeal K., Sanchez L., Zhang C., Stiles L., Zheng J.J. (2022). Characterizing the metabolic profile of dexamethasone treated human trabecular meshwork cells. Exp. Eye Res..

[21] Puca F., Yu F., Bartolacci C., Pettazzoni P., Carugo A., Huang-Hobbs E., Liu J., Zanca C., Carbone F., Del Poggetto E. (2021). Medium-Chain Acyl-CoA Dehydrogenase Protects Mitochondria from Lipid Peroxidation in Glioblastoma. Cancer Discov..

[22] Marcoccia R., Nesci S., Merlo B., Ballotta G., Algieri C., Pagliarani A., Iacono E. (2021). Biological characteristics and metabolic profile of canine mesenchymal stem cells isolated from adipose tissue and umbilical cord matrix. PLoS ONE.

[23] Duraj T., Carrión-Navarro J., Seyfried T.N., García-Romero N., Ayuso-Sacido A. (2021). Metabolic therapy and bioenergetic analysis: The missing piece of the puzzle. Mol. Metab..

[24] Gropman A., Uittenbogaard M., Brantner C.A., Wang Y., Wong L.-J., Chiaramello A. (2020). Molecular genetic and mitochondrial metabolic analyses confirm the suspected mitochondrial etiology in a pediatric patient with an atypical form of alternating hemiplegia of childhood. Mol. Genet. Metab. Rep..

[25] Chaphalkar R.M., Stankowska D.L., He S., Kodati B., Phillips N., Prah J., Yang S., Krishnamoorthy R.R. (2020). Endothelin-1 Mediated Decrease in Mitochondrial Gene Expression and Bioenergetics Contribute to Neurodegeneration of Retinal Ganglion Cells. Sci. Rep..

[26] Gaetani G.F., Galiano S., Canepa L., Ferraris A.M., Kirkman H.N. (1989). Catalase and glutathione peroxidase are equally active in detoxification of hydrogen peroxide in human erythrocytes. Blood.

[27] Alfonso-Prieto M., Biarnés X., Vidossich P., Rovira C. (2009). The Molecular Mechanism of the Catalase Reaction. J. Am. Chem. Soc..

[28] Palma F.R., Gantner B.N., Sakiyama M.J., Kayzuka C., Shukla S., Lacchini R., Cunniff B., Bonini M.G. (2024). ROS production by mitochondria: Function or dysfunction?. Oncogene.

[29] Chance B., Sies H., Boveris A. (1979). Hydroperoxide metabolism in mammalian organs. Physiol. Rev..

[30] Hernandez-Saavedra D., Swain K., Tuder R., Petersen S.V., Nozik-Grayck E. (2017). Redox Regulation of the Superoxide Dismutases SOD3 and SOD2 in the Pulmonary Circulation. Adv. Exp. Med. Biol..

[31] Hernansanz-Agustín P., Enríquez J.A. (2021). Generation of Reactive Oxygen Species by Mitochondria. Antioxidants.

[32] Becker S., L’Ecuyer Z., Jones B.W., Zouache M.A., McDonnell F.S., Vinberg F. (2024). Modeling complex age-related eye disease. Prog. Retin. Eye Res..

[33] Puigserver P., Adelmant G., Wu Z., Fan M., Xu J., O’Malley B., Spiegelman B.M. (1999). Activation of PPARgamma coactivator-1 through transcription factor docking. Science.

[34] Martínez-Redondo V., Pettersson A.T., Ruas J.L. (2015). The hitchhiker’s guide to PGC-1α isoform structure and biological functions. Diabetologia.

[35] Scarpulla R.C., Vega R.B., Kelly D.P. (2012). Transcriptional integration of mitochondrial biogenesis. Trends Endocrinol. Metab. TEM.

[36] Iacovelli J., Rowe G.C., Khadka A., Diaz-Aguilar D., Spencer C., Arany Z., Saint-Geniez M. (2016). PGC-1α Induces Human RPE Oxidative Metabolism and Antioxidant Capacity. Investig. Ophthalmol. Vis. Sci..

[37] St-Pierre J., Drori S., Uldry M., Silvaggi J.M., Rhee J., Jäger S., Handschin C., Zheng K., Lin J., Yang W. (2006). Suppression of reactive oxygen species and neurodegeneration by the PGC-1 transcriptional coactivators. Cell.

[38] Choi H.-I., Kim H.-J., Park J.-S., Kim I.-J., Bae E.H., Ma S.K., Kim S.W. (2017). PGC-1α attenuates hydrogen peroxide-induced apoptotic cell death by upregulating Nrf-2 via GSK3β inactivation mediated by activated p38 in HK-2 Cells. Sci. Rep..

[39] Virbasius J.V., Scarpulla R.C. (1994). Activation of the human mitochondrial transcription factor A gene by nuclear respiratory factors: A potential regulatory link between nuclear and mitochondrial gene expression in organelle biogenesis. Proc. Natl. Acad. Sci. USA.

[40] Hock M.B., Kralli A. (2009). Transcriptional control of mitochondrial biogenesis and function. Annu. Rev. Physiol..

[41] Xu W., Boyd R.M., Tree M.O., Samkari F., Zhao L. (2019). Mitochondrial transcription factor A promotes DNA strand cleavage at abasic sites. Proc. Natl. Acad. Sci. USA.

[42] Fuchshofer R., Tamm E.R. (2012). The role of TGF-β in the pathogenesis of primary open-angle glaucoma. Cell Tissue Res..

[43] Agarwal P., Daher A.M., Agarwal R. (2015). Aqueous humor TGF-β2 levels in patients with open-angle glaucoma: A meta-analysis. Mol. Vis..

[44] Fleenor D.L., Shepard A.R., Hellberg P.E., Jacobson N., Pang I.-H., Clark A.F. (2006). TGFbeta2-induced changes in human trabecular meshwork: Implications for intraocular pressure. Investig. Ophthalmol. Vis. Sci..

[45] Morgan J.T., Wood J.A., Walker N.J., Raghunathan V.K., Borjesson D.L., Murphy C.J., Russell P. (2014). Human trabecular meshwork cells exhibit several characteristics of, but are distinct from, adipose-derived mesenchymal stem cells. J. Ocul. Pharmacol. Ther. Off. J. Assoc. Ocul. Pharmacol. Ther..

[46] Keller K.E., Bhattacharya S.K., Borrás T., Brunner T.M., Chansangpetch S., Clark A.F., Dismuke W.M., Du Y., Elliott M.H., Ethier C.R. (2018). Consensus recommendations for trabecular meshwork cell isolation, characterization and culture. Exp. Eye Res..

[47] Clemente M.G., Patton J.T., Anders R.A., Yolken R.H., Schwarz K.B. (2015). Rotavirus Infects Human Biliary Epithelial Cells and Stimulates Secretion of Cytokines IL-6 and IL-8 via MAPK Pathway. BioMed Res. Int..

[48] Park B.-C., Tibudan M., Samaraweera M., Shen X., Yue B.Y.J.T. (2007). Interaction between two glaucoma genes, optineurin and myocilin. Genes Cells Devoted Mol. Cell. Mech..

[49] Ferretta A., Gaballo A., Tanzarella P., Piccoli C., Capitanio N., Nico B., Annese T., Di Paola M., Dell’aquila C., De Mari M. (2014). Effect of resveratrol on mitochondrial function: Implications in parkin-associated familiar Parkinson’s disease. Biochim. Biophys. Acta.

[50] Ye J., Coulouris G., Zaretskaya I., Cutcutache I., Rozen S., Madden T.L. (2012). Primer-BLAST: A tool to design target-specific primers for polymerase chain reaction. BMC Bioinform..

[51] Mookerjee S.A., Goncalves R.L.S., Gerencser A.A., Nicholls D.G., Brand M.D. (2015). The contributions of respiration and glycolysis to extracellular acid production. Biochim. Biophys. Acta.

[52] Mookerjee S.A., Nicholls D.G., Brand M.D. (2016). Determining Maximum Glycolytic Capacity Using Extracellular Flux Measurements. PLoS ONE.

[53] Mookerjee S.A., Gerencser A.A., Nicholls D.G., Brand M.D. (2017). Quantifying intracellular rates of glycolytic and oxidative ATP production and consumption using extracellular flux measurements. J. Biol. Chem..

[54] Preibisch S., Saalfeld S., Tomancak P. (2009). Globally optimal stitching of tiled 3D microscopic image acquisitions. Bioinformatics.

[55] Romero N., Swain P.M., Kam Y., Rogers G. (2018). Bioenergetic profiling of cancer cell lines: Quantifying the impact of glycolysis on cell proliferation. Cancer Res..

[56] Giddings E.L., Champagne D.P., Wu M.-H., Laffin J.M., Thornton T.M., Valenca-Pereira F., Culp-Hill R., Fortner K.A., Romero N., East J. (2021). Mitochondrial ATP fuels ABC transporter-mediated drug efflux in cancer chemoresistance. Nat. Commun..

[57] Fujiwara M., Tian L., Le P.T., DeMambro V.E., Becker K.A., Rosen C.J., Guntur A.R. (2019). The mitophagy receptor Bcl-2-like protein 13 stimulates adipogenesis by regulating mitochondrial oxidative phosphorylation and apoptosis in mice. J. Biol. Chem..

[58] Tsiriyotis C., Spandidos D.A., Sekeris C.E. (1997). The mitochondrion as a primary site of action of glucocorticoids: Mitochondrial nucleotide sequences, showing similarity to hormone response elements, confer dexamethasone inducibility to chimaeric genes transfected in LATK- cells. Biochem. Biophys. Res. Commun..

[59] Demonacos C.V., Karayanni N., Hatzoglou E., Tsiriyiotis C., Spandidos D.A., Sekeris C.E. (1996). Mitochondrial genes as sites of primary action of steroid hormones. Steroids.

[60] Luan G., Li G., Ma X., Jin Y., Hu N., Li J., Wang Z., Wang H. (2019). Dexamethasone-Induced Mitochondrial Dysfunction and Insulin Resistance-Study in 3T3-L1 Adipocytes and Mitochondria Isolated from Mouse Liver. Molecules.

[61] Liu J., Peng Y., Wang X., Fan Y., Qin C., Shi L., Tang Y., Cao K., Li H., Long J. (2016). Mitochondrial Dysfunction Launches Dexamethasone-Induced Skeletal Muscle Atrophy via AMPK/FOXO3 Signaling. Mol. Pharm..

[62] Pandya J.D., Agarwal N.A., Katyare S.S. (2004). Effect of dexamethasone treatment on oxidative energy metabolism in rat liver mitochondria during postnatal developmental periods. Drug Chem. Toxicol..

[63] Roussel D., Dumas J.-F., Augeraud A., Douay O., Foussard F., Malthiéry Y., Simard G., Ritz P. (2003). Dexamethasone treatment specifically increases the basal proton conductance of rat liver mitochondria. FEBS Lett..

[64] Sharma A., Patil A.J., Mansoor S., Estrago-Franco M.F., Raymond V., Kenney M.C., Kuppermann B.D. (2013). Effects of dexamethasone on human trabecular meshwork cells in vitro. Graefes Arch. Clin. Exp. Ophthalmol. Albrecht Graefes Arch. Klin. Exp. Ophthalmol..

[65] Filla M.S., Schwinn M.K., Nosie A.K., Clark R.W., Peters D.M. (2011). Dexamethasone-associated cross-linked actin network formation in human trabecular meshwork cells involves β3 integrin signaling. Investig. Ophthalmol. Vis. Sci..

[66] Faralli J.A., Gagen D., Filla M.S., Crotti T.N., Peters D.M. (2013). Dexamethasone increases αvβ3 integrin expression and affinity through a calcineurin/NFAT pathway. Biochim. Biophys. Acta.

[67] Psarra A.-M.G., Sekeris C.E. (2011). Glucocorticoids induce mitochondrial gene transcription in HepG2 cells: Role of the mitochondrial glucocorticoid receptor. Biochim. Biophys. Acta.

[68] Hunter R.G., Seligsohn M., Rubin T.G., Griffiths B.B., Ozdemir Y., Pfaff D.W., Datson N.A., McEwen B.S. (2016). Stress and corticosteroids regulate rat hippocampal mitochondrial DNA gene expression via the glucocorticoid receptor. Proc. Natl. Acad. Sci. USA.

[69] Weber K., Brück P., Mikes Z., Küpper J.-H., Klingenspor M., Wiesner R.J. (2002). Glucocorticoid hormone stimulates mitochondrial biogenesis specifically in skeletal muscle. Endocrinology.

[70] Sibayan S.A., Latina M.A., Sherwood M.E., Flotte T.J., White K. (1998). Apoptosis and morphologic changes in drug-treated trabecular meshwork cells in vitro. Exp. Eye Res..

[71] Watanabe M., Sato T., Tsugeno Y., Umetsu A., Suzuki S., Furuhashi M., Ida Y., Hikage F., Ohguro H. (2022). Human Trabecular Meshwork (HTM) Cells Treated with TGF-β2 or Dexamethasone Respond to Compression Stress in Different Manners. Biomedicines.

[72] Xie L., Feng H., Li S., Meng G., Liu S., Tang X., Ma Y., Han Y., Xiao Y., Gu Y. (2016). SIRT3 Mediates the Antioxidant Effect of Hydrogen Sulfide in Endothelial Cells. Antioxid. Redox Signal..

[73] Schiffmann L.M., Werthenbach J.P., Heintges-Kleinhofer F., Seeger J.M., Fritsch M., Günther S.D., Willenborg S., Brodesser S., Lucas C., Jüngst C. (2020). Mitochondrial respiration controls neoangiogenesis during wound healing and tumour growth. Nat. Commun..

[74] Marchetti P., Fovez Q., Germain N., Khamari R., Kluza J. (2020). Mitochondrial spare respiratory capacity: Mechanisms, regulation, and significance in non-transformed and cancer cells. FASEB J. Off. Publ. Fed. Am. Soc. Exp. Biol..

[75] Willems P.H.G.M., Rossignol R., Dieteren C.E.J., Murphy M.P., Koopman W.J.H. (2015). Redox Homeostasis and Mitochondrial Dynamics. Cell Metab..

